# Combined Liver and Kidney Transplant in a Patient with Budd-Chiari Syndrome Secondary to Autosomal Dominant Polycystic Kidney Disease Associated with Polycystic Liver Disease: Report of a Case with a 9-Year Follow-Up

**DOI:** 10.1155/2014/585291

**Published:** 2014-06-01

**Authors:** Patricia Ramírez de la Piscina, Ileana Duca, Silvia Estrada, Rosario Calderón, Idoia Ganchegui, Amaia Campos, Katerina Spicakova, Leire Urtasun, Marta Salvador, Elvira Delgado, Raquel Bengoa, Francisco García-Campos

**Affiliations:** Department of Gastroenterology, Hospital Txagorritxu, Universidad del País Vasco, C/ José Achotegui s/n, Vitoria-Gasteiz, 01009 Álava, Spain

## Abstract

Polycystic liver disease (PLD) is a hereditary disease inherited by autosomal dominant trait that occurs as a frequent extrarenal manifestation of autosomal dominant polycystic kidney disease (ADPKD). We report a case of a 59-year-old woman diagnosed with ADPKD associated with PLD. End-stage chronic renal failure with a secondary Budd-Chiari syndrome developed during the patient's clinical course. She underwent combined liver and kidney transplantation, with a successful response over a 9-year follow-up period.

## 1. Introduction

Autosomal dominant polycystic kidney disease (ADPKD) is characterized by the formation of renal cysts and a variety of extrarenal manifestations of which polycystic liver disease (PLD) is the most common. It is caused by mutations in one of two genes,* PKD1* or* PKD2* [1, 2]. ADPKD affects up to 0.2% of the general population [3]. On the other hand, isolated PLD has prevalence of less than 0.01% [4, 5].

Liver cysts are the most frequent extrarenal manifestations of ADPKD. The prevalence of liver cysts is between 75 to 90% among ADPKD patients [6].

In the PLD the formation of liver cysts arises from cholangiocytes. Cysts further expand due to various processes, such as cell matrix interactions, cell proliferation, apoptosis and differentiation, and fluid secretion [7]. Different factors could impact this process and promote cysts growth. One of these factors is cAMP which is involved in the regulation of cellular processes, for example, in the liver cyst formation. cAMP also plays a significant role in kidney cyst growth and reduction of cAMP levels* in vivo* attenuates renal progression.

Liver cysts usually appear later than kidney cysts, often during the fourth or fifth decade of life, and are more common in women. It is believed that female steroid hormones may influence several of the factors responsible for growth of liver cysts [8]. The number and volume of renal cysts are correlated with age, severity of kidney disease, and decline of glomerular filtration rate. Structural and functional renal deterioration occurs in ADPKD patients and is the fourth leading cause of end-stage renal disease in adults [9].

Most patients with PLD remain asymptomatic and the diagnosis is usually incidental associated with elevation of cytolysis-related enzymes, gamma-glutamyl transpeptidase (*γ*-GGT), and alkaline phosphatase detected at a routine laboratory study [10]. However, the large bulky cysts within the abdomen may cause abdominal distension, early postprandial fullness and abdominal discomfort, painful hepatomegaly, or lumbar pain. The management of patients with symptomatic PLD is challenging. There are three types of treatments: medical, interventional radiology, and surgery.

Although there are no approved medical treatments for PLD, somatostatin analogues and mammalian target of rapamycin inhibitors are promising therapies [11].

Somatostatin analogues are inhibitors of cAMP, so they can reduce the secretion of fluid and cholangiocytes proliferation. Two randomized controlled trials have demonstrated that, after 6 to 12 months of treatment with lanreotide, a significant reduction of liver volume was achieved, compared with placebo [12, 13]. However, the average liver volume reduction was only 3% to 5% and the abdominal symptoms were not significantly improved. So this treatment might be used only in a selected group of patients with symptomatic PLD in whom the risks of surgical intervention are not justified or it is technically challenging.

On the other hand, mammalian target rapamycin (m-Tor) inhibitors have antiproliferative and immunosuppressive effects, but although some studies in animal models [11] appeared to have promising results, others did not show substantial therapeutic effects neither in humans [14] nor in animal models [15].

Among interventional and surgical treatment options, the most common are percutaneous cyst aspiration, laparoscopic or open cyst fenestration, hepatic resection, and transplantation. Resection and transplantation are technically difficult in these patients due to massive size of the liver and distortion of usual anatomic landmarks [8]. Ascites and variceal bleeding are uncommon in the ADPKD patient with liver cysts, but when present investigation to rule out vascular thrombosis is indicated [16]. Huge liver cysts may cause hepatic venous outflow obstruction with a secondary Budd-Chiari syndrome [17]. In this context, and in the presence of ESRD, the patient may be a candidate for combined liver and kidney transplantation.

We here report the case of a 59-year-old woman diagnosed with ADPKD and end-stage renal disease who developed a secondary Budd-Chiari syndrome and was successfully treated with combined liver and kidney transplantation. At present, after 9 years of follow-up, the patient remained asymptomatic with complete resumption of daily life activities. This case draws attention to the possibility of a double liver and kidney transplant for the management of ADPKD patients with liver cysts complicated by the development of a Budd-Chiari syndrome.

## 2. Case Report

**Figure 1:**
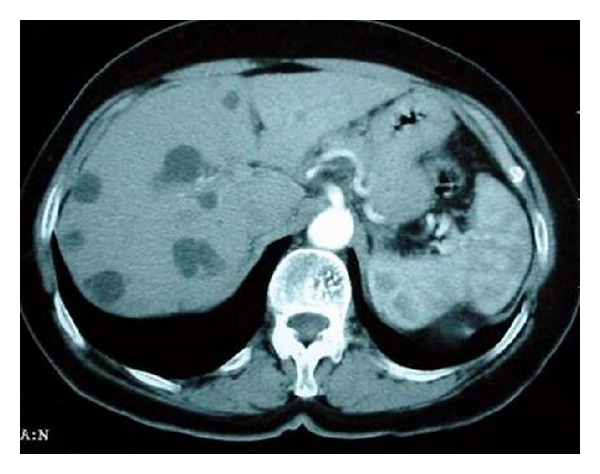
Abdominal and pelvic CT scan in a patient with hepatorenal polycystic disease. Large hepatic cysts causing stenosis and displacement of the inferior vena cava and compression of the suprahepatic veins in relation to a Budd-Chiari syndrome.

A 59-year-old woman was diagnosed at the age of 37 with hypertension and renal insufficiency secondary to ADPKD associated with PLD, requiring treatment with peritoneal dialysis. Fourteen years after diagnosis, she was admitted to the hospital with asthenia, abdominal distension, hypotension, and oligoanuria. Painful hepatomegaly (3 cm below the costal margin) was present. Salient findings of laboratory tests included serum creatinine of 7.4 mg/dL, serum albumin 2.6 g/dL, alanine aminotransferase (ALT) 109 IU/L, aspartate aminotransferase (AST) 94 IU/L, *γ*-GGT 24 IU/L, alkaline phosphatase 72 IU/L, and platelet count 93 × 10^9^/L. Other examinations for liver-related conditions were negative or within normal ranges, such as antinuclear, antimitochondrial, and antismooth muscle antibodies, porphyrin tests, ceruloplasmin, alpha1-antitrypsin, thyroid hormones, anti-transglutaminase, iron profile, and serological tests for hepatitis B and hepatitis C virus infection. Multiple cysts occupying almost the entire hepatic and renal parenchyma were detected in the abdominal computed tomography scan (TC) (Figure 1). Liver cysts were of large size causing stenosis and displacement of the superior vena cava. The suprahepatic veins were not visualized and thrombosis could not be excluded. Also, patchy uptake of the contrast by the liver parenchyma with hypodense areas in the left hepatic lobe was found. These findings together with the presence of ascitic fluid supported the clinical suspicion of a Budd-Chiari syndrome. An abdominal Doppler ultrasound study revealed stenosis of the inferior vena cava, with patent suprahepatic veins in association with loss of the morphology of the triphasic waves. Stenosis of the inferior vena cava by extrinsic compression due to multiple hepatic cysts was confirmed by inferior vena cavography and hepatic venography through puncture of the right femoral vein. An upper gastrointestinal endoscopic examination showed esophageal varices grade I/III in the distal esophagus. A diagnostic paracentesis of ascitic fluid was performed and a high SAAG gradient (>1.1 g/dL) without data of bacterial peritonitis was obtained.

In the presence of ADPKD and PLD in a patient with end-stage renal disease, long-standing peritoneal dialysis, and multiple hepatic cysts with secondary Budd-Chiari syndrome, a combined liver and kidney transplantation was indicated. The patient underwent orthotopic liver transplantation with total hepatectomy and preservation of the inferior vena cava (piggyback technique). A cadaveric kidney transplant with the graft placed in the right iliac fossa was also performed. The postoperative course was uneventful. At the present time, after 9 years of follow-up, the patient remained asymptomatic with complete resumption of daily life activities. Current treatment includes tacrolimus (2 mg/day), mycophenolate mofetil (500 mg administered twice a day), calcitriol (0.25 mcg/day), and atorvastatin (10 mg/day).

## 3. Discussion

This case illustrates that, in the presence of ADPKD, with large multiple cysts in the liver and severe renal failure undergoing continuous ambulatory peritoneal dialysis, combined liver and kidney transplantation is a feasible alternative. In addition, our patient had secondary Budd-Chiari syndrome, the treatment of which with transjugular intrahepatic portosystemic shunt (TIPS) was not considered appropriate given the presence of end-stage renal disease. Resection of the liver cysts is also associated with significant morbidity and even mortality, and in cases of extensive hepatic cystic disease, liver transplantation may be the best option. Although the early mortality rate from performing the operation ranges from 10% to 20% [8], once patients recovered from the first 3-month postoperative period, their long-term survival after transplantation is excellent [18, 19]. Many patients with polycystic disease who choose to undergo transplantation are quite pleased, as it resolves most if not all of their symptoms [20, 21]. In the European Liver Transplant Registry (January 1988 to June 2001), PLD accounts for about 0.5% of the diagnoses for liver transplantation [22].

The patient characteristics in this study were comparable to those previously reported, mostly middle-aged women with painful hepatomegaly as the most common symptom and, in 34–78% of the cases, with concomitant polycystic kidney disease [23].

In relation to the pathogenesis of hepatic cysts in ADPKD, it has been suggested that both ductal-plate malformation due to alteration of apoptosis process and hyperproliferation of cholangiocytes as consequence of the altered balance between fluid secretion and absorption in the lumen of the biliary ducts are due to an increased cytoplasmatic level of cAMP [6, 7]. Although there are no approved medical treatments for PLD, somatostatin analogues and mammalian target of rapamycin inhibitors are promising therapies [11].

Somatostatin analogues are inhibitors of cAMP, so they can reduce the secretion of fluid and although some studies have demonstrated somatostatin benefit, it is limited so this treatment might be used only in a selected group of patients with symptomatic PLD in whom the risks of surgical intervention are not justified or it is technically challenging.

On the other hand, mammalian target rapamycin (m-Tor) inhibitors have antiproliferative and immunosuppressive effects, but although some studies in animal models [11] appeared to have promising results, others did not show substantial therapeutic effects neither in humans [14] nor in animal models [15].

It is rare for PLD patients to present with specific liver problems such as portal hypertension, ascites, or variceal bleeding. Patients with liver cysts can also experience impingement on the venous drainage of the liver, causing a secondary Budd-Chiari syndrome, which blocks the venous drainage from the liver [8]. In our patient, the Budd-Chiari syndrome was related to compression of the inferior vena cava by the hepatic cysts. However, Budd-Chiari syndrome in association with ADPKD due extrinsic compression of the hepatic veins and the inferior vena cava by liver cysts has been rarely reported [17, 24–26]. Budd-Chiari syndrome can also develop as a complication of nephrectomy in patients with liver cysts [27].

In summary, we here report a case of Budd-Chiari syndrome as a complication of liver cysts in a patient with ADPKD. Combined liver and kidney transplantation was chosen as a treatment option for PLD and progression of the renal disease requiring peritoneal dialysis maintenance treatment. This is the first report of a patient with hepatorenal polycystic disease that presented a secondary Budd-Chiari syndrome due to extrinsic compression of the inferior vena cava, successfully treated with combined liver and kidney transplantation with excellent results after a follow-up of 9 years.
